# Regulatory Role of Small Nucleolar RNAs in Human Diseases

**DOI:** 10.1155/2015/206849

**Published:** 2015-04-28

**Authors:** Grigory A. Stepanov, Julia A. Filippova, Andrey B. Komissarov, Elena V. Kuligina, Vladimir A. Richter, Dmitry V. Semenov

**Affiliations:** ^1^Institute of Chemical Biology and Fundamental Medicine, Siberian Branch of Russian Academy of Sciences, Lavrentiev Avenue 8, Novosibirsk 630090, Russia; ^2^Novosibirsk State University, Pirogova Street 2, Novosibirsk 630090, Russia; ^3^Research Institute of Influenza, Prof. Popova Street 15/17, Saint Petersburg 197376, Russia; ^4^Saint Petersburg State University, Universitetskaya Embankment, 7-9, Saint Petersburg 199034, Russia

## Abstract

Small nucleolar RNAs (snoRNAs) are appreciable players in gene expression regulation in human cells. The canonical function of box C/D and box H/ACA snoRNAs is posttranscriptional modification of ribosomal RNAs (rRNAs), namely, 2′-O-methylation and pseudouridylation, respectively. A series of independent studies demonstrated that snoRNAs, as well as other noncoding RNAs, serve as the source of various short regulatory RNAs. Some snoRNAs and their fragments can also participate in the regulation of alternative splicing and posttranscriptional modification of mRNA. Alterations in snoRNA expression in human cells can affect numerous vital cellular processes. SnoRNA level in human cells, blood serum, and plasma presents a promising target for diagnostics and treatment of human pathologies. Here we discuss the relation between snoRNAs and oncological, neurodegenerative, and viral diseases and also describe changes in snoRNA level in response to artificial stress and some drugs.

## 1. Introduction

Small nucleolar RNAs (snoRNAs) represent a class of regulatory RNAs responsible for posttranscriptional maturation of ribosomal RNAs (rRNAs). Small nucleolar RNAs are divided into two families based on their structure and main function: box C/D snoRNAs and box H/ACA snoRNAs. Box C/D RNAs are responsible for 2′-O-methylation, while the latter family guides pseudouridylation of nucleotides [1, 2].

**Figure 1:**
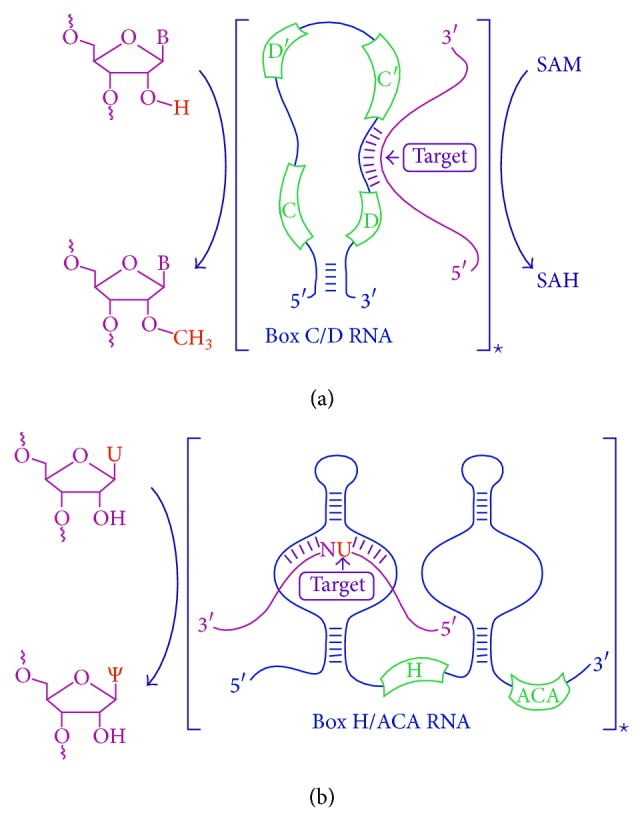
(a) Box C/D snoRNAs share two conserved elements: boxes С (PuUGAUGA) and D (CUGA) located at the 5′- and 3′-termini of the RNA molecule, respectively. Often, box C/D snoRNAs also have an additional copy of internally located C′ or D′ boxes. A complex of box C/D snoRNA with nucleolar proteins Nop1p (fibrillarin), Nop56p, Nop58p, and Snu13p (15.5 kDa) catalyzes site-specific 2′-O-methylation of the nucleotide in target RNA. S-Adenosylmethionine (SAM) serves as the donor of the methyl group and is converted to S-adenosylhomocysteine (SAH). (b) Box H/ACA snoRNAs have “hairpin-hinge-hairpin-tail” structure with boxes H (ANANNA) and ACA located within the single-stranded (hinge) and 3′-terminus (tail) regions, respectively. Pseudouridylation complex includes dyskerin (the pseudouridine synthase), GAR1, NHP2, and NOP10 proteins. Box H/ACA snoRNP catalyzes site-specific isomerization of uridine (U) to pseudouridine (Ψ) in target RNA. ∗SnoRNA with target RNA in square brackets designates snoRNP enzyme-substrate complex.

Eukaryotic box C/D RNAs are typically 70–120 nt in length and contain two conserved elements: boxes С (PuUGAUGA) and D (CUGA) located at the 5′- and 3′-termini of the RNA molecule, respectively (Figure 1(a)). These sequence elements form so-called “kink-turn” (stem-bulge-stem) structure that serves as the scaffold for the assembly of a small nucleolar ribonucleoprotein (snoRNP) including the following proteins: Nop1p (also known as fibrillarin), Nop56p, Nop58p, and Snu13p (15.5 kDa) [3–6]. Methyltransferase fibrillarin serves as the key component of snoRNPs: it catalyzes the transfer of a methyl group from S-adenosylmethionine (SAM) to 2′-O-position of the target nucleotide. The methylation guide sequence exhibits complementarity to the region of the target RNA; it is 10–21 nt long and located upstream of the box D/D′ motifs. The base in the target RNA paired to the fifth nucleotide upstream of the box D/D′ sequence is to be methylated [1, 7].

Box H/ACA family snoRNAs share hallmark secondary structure called “hairpin-hinge-hairpin-tail” that includes two hairpin domains linked with a single-stranded region (hinge) and 3′-terminus region (tail) [2]. Boxes H and ACA are located in a close vicinity of the hairpin in the hinge and tail regions, respectively (Figure 1(b)). Box H represents a conserved ANANNA (N stands for any nucleotide) motif, while box ACA is a trinucleotide located 3 nt before the 3′-terminus. Each hairpin consists of internal and external loops. A 9-13-nt-long guide sequence is located on both strands of the inner loop. During interaction with the target RNA molecule the complementary sequences of box H/ACA RNA flank two nucleotides including a uridine residue, which is further subjected to U/Ψ isomerization. Thus, the three-dimensional structure of the conserved motif enables the access of pseudouridylation enzymes to the substrate uridine. The distance between the target nucleotide and H/ACA box in rRNA-snoRNA duplex is 14-15 nt long [2, 8].

Similar elements, boxes C and D as well as boxes H and ACA, can be found in primary structures of small Cajal body-specific RNAs (scaRNAs). These RNAs guide 2′-O-methylation and pseudouridylation of small nuclear RNAs (snRNAs) [9, 10].

It has been shown that numerous box C/D and box H/ACA RNAs have no identified targets among rRNAs or snRNAs. Such snoRNAs, referred to as “orphan snoRNAs/scaRNAs,” may have some other regulatory function aside from 2′-O-methylation and pseudouridylation of RNA nucleotides [11–13]. Series of studies of snoRNA expression profiles in mammalian cells have demonstrated that the level of many “orphan” box C/D and box H/ACA RNAs varies among different tissues [14–17]. The variation of snoRNA expression profiles suggests that such RNAs have tissue-specific functions. For example, two brain-specific box C/D RNAs SNORD115 (HBII-52) and SNORD116 (HBII-85) were shown to be processed into smaller RNAs that can affect the outcome of alternative splicing of various pre-mRNAs by preventing the formation of particular pre-mRNA splicing variants [18]. SNORD115 is also involved in the regulation of serotonin receptor 5-HT2CR mRNA level in brain cells through alternative splicing and control of posttranscriptional nucleotide modification (adenosine-to-inosine editing) of the target mRNA [19, 20].

Along with SNORD115 and SNORD116, a number of other human snoRNAs can generate smaller fragments, as it has been demonstrated by high-throughput sequencing and RNase protection experiments [21–23]. Short fragments derived from box C/D snoRNA SNORD88C (HBII-180C) hold complementarity to fibroblast growth receptor-3 (FGFR-3) pre-mRNA. Being expressed from artificial “snoMEN” vector, SNORD88C (HBII-180C) is capable of suppressing target gene mRNA and protein level in a sequence-dependent manner through base pairing with pre-mRNA regulatory elements [24, 25]. Bioinformatic analysis showed that about a half of all box C/D RNAs are processed into small molecules that are conserved among multiple cell lines [26]. Box H/ACA snoRNAs are usually processed into 20–24 nt fragments, while box C/D-derived RNAs are ~17–19 or >24 nt long. These fragments are termed snoRNA-derived RNAs (sdRNAs) and considered to be a novel class of regulatory RNAs. Most of sdRNAs preserve conserved C/D or H/ACA box pairs [21, 27, 28]. Moreover, several sdRNAs were found to interact with Ago family proteins, the key components of RISC complexes [29, 30]. It was shown that a number of box C/D RNA-derived fragments can exhibit miRNA activity [28]. However, the mechanism underlying the biogenesis of such snoRNA derivatives that resemble microRNAs still remains unknown. It has been shown that processing of box C/D RNAs into sdRNAs was Dicer-independent, while production of box H/ACA-derived sdRNAs required Dicer [29, 31]. Thus, snoRNAs can serve as a source of short regulatory RNA species involved in the control of processing and translation of various mRNAs.

Each of the functions described above may be implicated in the initiation and progression of pathology. The study of snoRNA function will have significant diagnostic and therapeutic value. This review deals with the latest data on the relationship between snoRNAs and socially important diseases such as oncological and neurodegenerative disorders. Changes in snoRNA level in mammalian cells subjected to stress and drugs are also described.

## 2. snoRNAs and Cancer

The defects in ribosome maturation and function can cause disruption of vital processes and lead to diseases and transformation of normal cells into tumor cells [32–34]. Since snoRNAs are involved in the regulation of posttranscriptional modification of ribosomal RNAs, it is fair to assume that snoRNA level can affect physiological condition of cells, tissues, and organs. Thus, the change in snoRNA expression level may lead to various diseases. Hence, the studies aimed at evaluating snoRNA expression levels in human and mammalian cells might underpin the development of diagnostic approaches and generation of novel therapeutic agents.

Research in this field is mostly focused on the involvement of human snoRNAs in oncological diseases. In mammalians the majority of snoRNAs are encoded within the introns of protein coding or noncoding genes, so-called “host-genes” (reviewed in [35, 36]). Therefore, an alteration of snoRNA expression may result from the oncogenic processes accompanied by changes in transcriptional activity of the host-gene or aberrations of nuclear maturation of the pol II transcript. In that case, the snoRNA, cellular level of which rises/drops rapidly, may not play a key role in the pathology, but the alteration itself may indicate tumor progression and reflect metastatic potential [37]. On the other hand, snoRNA, the expression of which is positively or negatively correlated with tumor cells proliferation and invasion, may have considerable impact on carcinogenesis and thus should be treated as an oncogene when its expression goes far beyond the “normal level” [38].

### 2.1. The Role of snoRNAs in Oncotransformation of Mammalian Cells

Specific increase in the expression level of several box C/D RNAs was detected in murine and human breast cancer cells [39]. Both breast cancer primary cultures and breast cancer cell lines (MCF-7 and ZR-75-1) exhibited increased level of U22, U3, U8, U94, and U97 box C/D snoRNAs compared to normal and untransformed (MCF-10A) human cells. Change in snoRNA expression level is accompanied by an increase in fibrillarin, the core component of snoRNPs, with its level correlating with the expression of* Myc* oncogene. High level of fibrillarin was observed not only in breast cancer cells, but also in prostate and many other human cancers. Targeted repression of fibrillarin and other proteins of the methyltransferase complex (NOP56 and NOP58) not only led to the decrease in box C/D snoRNA level, but also inhibited cancer cell growth and decreased oncogenicity [39]. siRNA-mediated knockdown of fibrillarin in cancer cells enhanced induction of tumor suppressor p53 protein. Meanwhile, increased fibrillarin expression resulted in significantly compromised p53-dependent response to stress in cancer cells. Earlier, it has been demonstrated that disrupted nucleolar activity, such as blocked transcription of rRNA genes, leads to p53 protein stabilization due to the binding between MDM2 (negative regulator of p53) and ribosomal proteins (RPL5, RPL11, and RPL23) [40]. Thus, the presented data elucidate the role of nucleolar components, including snoRNAs and snoRNP proteins, in survival of cancer cells by modulation of p53 expression.

Small nucleolar box H/ACA RNA SNORA42, which is overexpressed in nonsmall cell lung carcinoma (NSCLC), was identified as a lung cancer oncogene [38]. siRNA knockdown of SNORA42 results in reduced cancer cell growth (H460 and H1944 cells), inhibited H460 and H1944 cell colony formation, and compromised tumorigenicity in animal models. Specific SNORA42 suppression in cancer cells leads to apoptosis in a p53-dependent manner. On the contrary, overexpression of SNORA42 activates cancer cell growth. The expression level of SNORA42 in lung tumor tissue specimen was found to be inversely correlated with the survival of NSCLC patients [38].

ACA11 is a peculiar snoRNA encoded within an intron of* WHSC1* that exhibits the structure of a box H/ACA RNA and is predominantly localized in the nucleolus [41]. However, it has been demonstrated that it does not associate with dyskerin and other components of the pseudouridylation machinery typical of H/ACA snoRNPs. Instead, it binds to the proteins implicated in RNA processing: RNA splicing factors (SF3B1, SF3B2, and SFPQ), ATP-dependent RNA helicase A (DHX9), RNA-specific adenosine deaminase (ADAR), and the members of the heterogeneous nuclear ribonucleoprotein family. ACA11 is highly expressed in multiple myeloma, colon, esophageal, and bladder cancers. ACA11 overexpression was found to induce downregulation of ribosomal protein genes and snRNA involved in oxidative stress regulation, resulting in resistance to chemotherapy and enhancing multiple myeloma cell proliferation [41].

Studies in mouse models have uncovered that adeno-associated virus integrates with high-frequency on chromosome 12 near* Meg3* gene locus [42]. This region is rich in orphan snoRNA and microRNA genes. The integration of the viral genome causes aberrations in host-gene expression leading to hepatocellular carcinoma. The syntenic region in humans is proposed to encode tumor suppressor genes. The expression of the noncoding* Meg3* transcript is severely suppressed in various cancer cell lines and tumor types, while the deletion of the corresponding genome locus leads to aggressive tumors [43].* Meg3* RNA upregulates p53 levels by downregulating Mdm2 levels and promotes angiogenesis by increasing Vegfa and Vegfr1 levels [44, 45].

Two other genes* ZFAS1* and* GAS5*, which host snoRNAs within their introns, have been associated with numerous types of cancers [37, 46–49].* GAS5* locus contains 10 box С/D RNAs that guide modification of various nucleotides in 18S and 28S rRNAs. The expression of two* GAS5*-associated snoRNAs U44 and U47 was found upregulated in colorectal tumors compared to benign colon tissues [46]. Meanwhile, in breast cancer, head and neck squamous cell carcinoma (SCC) low expression level of U44 directly correlated with poor survival of patients [37, 47].* ZFAS1* contains three box С/D RNAs: SNORD12, SNORD12B, and SNORD12C, that guide 2′-O-methylation of G3878 in 28S rRNA.* ZFAS1* locus and these snoRNAs were shown to be strongly downregulated in breast ductal carcinoma [49].

SnoRNA expression level was found decreased in acute myeloid leukemia (AML) and acute lymphoblastic leukemia (ALL) cells, compared to normal cells [50]. Underexpression has been reported for another class of ncRNAs, microRNAs, in several types of cancers [50, 51]. However, a few snoRNAs are overexpressed in some AML cases. Specific overexpression has been reported for several orphan box C/D RNAs: SNORD112-114, encoded within the DLK1-Dio3 locus of chromosome 14. SNORD114-1 has been shown to promote cell cycle progression through G0/G1 to S phase transition [50]. Overexpression of SNORD114-1 activates proliferation in K562 cell line, while targeted suppression of the box C/D RNA leads to the induction of cell death. The data implicate that some of these box C/D RNAs participate in the development of leukemia.

One of the mechanisms regulating overall level of snoRNA in cancer cells may be the methylation of the CpG islands located close to snoRNA genes. The DNA methylation status of CpG islands within 2 kb from 5′-termini of 49 snoRNA genes was analysed in HCT-116 colorectal cancer cells [52]. Hypermethylation of the CpG islands, located near the 5′-termini of host-genes for SNORD123, SNORA70C, and SNORA59B, was established to be specific for cancer cells, compared to normal human cells. Further analysis enabled us to reveal similar DNA methylation profile close to these snoRNA host-genes in melanoma, lymphoma and leukemia cell lines, lung, breast, prostate, ovary, and kidney cancers, as well as the primary cultures of blood cells in patients with ALL. Furthermore, the expression of these box C/D and H/ACA snoRNAs in HCT-116 and SW48 cells was downregulated, compared to the cells lacking CpG island methylation near SNORD123, SNORA70C, and 59B snoRNA host-genes. The result obtained suggests the existence of epigenetic control mechanism for the regulation of snoRNA expression, which might be common for various cancer cells. The expression of other noncoding RNA genes is also known to be regulated by changing the methylation status in adjacent chromosome regions. For example, CpG island methylation in cancer cells can result in the repression of microRNAs that are reported to be tumor suppressors [53].

Martens-Uzunova et al. demonstrated that some box C/D and box H/ACA snoRNAs, as well as several tRNAs, are overexpressed in prostate cancer cells during the premetastatic stages [54]. The data claim that snoRNAs are capable of active participation in tumor progression. Ribosomal RNA biogenesis is known to be more robust in cancer cells than in normal cells [55]. Therefore, it can be assumed that the increase in snoRNAs level is necessary for the acceleration of rRNA maturation, ribosome assembling, and protein synthesis during tumorigenesis. It should be noted that the relationship between the deregulation of snoRNA expression and rRNA biogenesis in diseases still remains elusive. Further studies in snoRNA research area should be addressed to this issue.

### 2.2. Oncodiagnostic Value of snoRNAs

In a recent study, Liao et al. have profiled the expression signatures of 352 snoRNAs in NSCLC [56]. Six snoRNAs—SNORD33, SNORD66, SNORD73B, SNORD76, SNORD78 and SNORA42—were established to be overexpressed both in adenocarcinoma (AC) and SCC [56]. All 6 snoRNAs are present in a stable form in human blood plasma, and their level can be accurately measured by RT-qPCR. A new diagnostic system with 81.1% sensitivity and 95.8% specificity was proposed based on simultaneous assessment of three snoRNAs (SNORD33, SNORD66, and SNORD76) blood plasma levels. According to the data presented, the usage of the three diagnostic genes allows us not only to detect NSCLC, but also to distinguish it from chronic obstructive pulmonary disease. However, the levels of SNORD33, SNORD66, and SNORD76 do not reflect the stage of malignancy or histological origin [56].

A homozygous 2 bp (TT) deletion in U50 snoRNA gene, encoded within the intron of* U50HG* (*SNHG5*), was found in prostate cancer cell lines [57]. The same, but often heterozygous, 2-nt mutation in* U50HG* (*SNHG5*) gene is present in breast cancer cells [58]. The deletion leads to the reduced expression of U50 snoRNA. In both cases the return to the wild-type expression level of the snoRNA results in inhibited cancer cell growth. The mutation was detected with diagnostically significant frequencies in blood cells of males with prostate cancer and females with breast cancer [57, 58]. The data allowed identifying U50 box C/D RNA as a tumor suppressor.

Expression profile of snoRNA and scaRNA genes was analyzed with quantitative RT-PCR in leukemic cells of different origin—AML, T-ALL and pre-B ALL, Burkitt's lymphoma/leukemia, and a solid tumor (HeLa cell line) [59]. Box C/D and box H/ACA snoRNA expression profiles were found to vary within different types and subtypes of cancer, with different leukemia subtypes sharing the most similarity. That difference in snoRNA levels enables us to use snoRNA and scaRNA patterns for characterization and classification of cancer types [59]. Diagnostically useful differences in snoRNA expression profiles were also reported for different subtypes of T-cell lymphoma [60]. Elevated level of box C/D RNA HBII-239 and its microRNA derivative miRNA-768-3p in peripheral T-cell lymphoma was detected in cases with favorable outcomes.

In conclusion, the data reported suggest that there are meaningful changes in box C/D and box H/ACA snoRNA levels in human cells and body fluids during the development of oncological diseases. Small nucleolar RNAs were suggested both as biomarkers of oncological diseases and as targets for cancer therapy. Other ncRNAs, first of all circulating in human blood plasma microRNAs, have been considered to be perspective minimally invasive markers of diseases [61–66]. Thus, small nucleolar RNAs supplement the set of therapeutically and diagnostically significant regulatory RNAs, and the fundamental knowledge of snoRNA structure and biological functions can be applied to the development of new therapeutic approaches for cancer treatment.

## 3. snoRNAs and Human Neurodegenerative Disorder

The relation between box C/D RNAs and neurodegenerative disease development has been also described [67, 68]. In a series of independent studies the genetic disorder, Prader-Willi syndrome (PWS), was shown to be caused by the loss of paternal gene expression from a maternally imprinted region 15q11–q13 on chromosome 15. The disease is featured by mental retardation, low height, obesity, and muscle hypotonia. Locus 15q11–q13 contains numerous copies of two box C/D RNAs—SNORD115 (HBII-52) and SNORD116 (HBII-85) [16]. Box C/D RNA SNORD115 may have an impact on 5-HT2CR serotonin receptor mRNA level in brain [19, 20]. The loss of the SNORD116 snoRNAs can be a significant contribution to the etiology of PWS [16, 69, 70].

The deletion of PWS critical region that contains the* SNURF-SNRPN* locus, as it has been demonstrated in mouse model of PWS, leads to the changes in splicing patterns of several pre-mRNAs [18]. The data also indicates that SNORD115, as well as SNORD116, undergoes specific hydrolysis forming smaller fragments, processed snoRNAs referred to as “psnoRNAs” [18, 22]. These processed species of SNORD115, but not the full-length snoRNA, were suggested to affect the outcome of alternative splicing of the pre-mRNAs [18]. However, Bortolin-Cavaillé and Cavaillé provided evidence against the existence of abundant processed variants of SNORD115 and SNORD116 (psnoRNAs) in human or mouse brain, concluding that PWS-encoded snoRNAs represent canonical box C/D snoRNAs [71].

A recent study revealed that the change in box C/D RNA level also takes place in brain cells during abnormal fetal development caused by maternal alcohol consumption during pregnancy [72]. Mouse model experiments demonstrated that, along with the change in DNA methylation pattern, brain tissue also exhibited changes in expression levels of some microRNAs and snoRNAs. Particularly, the increase in SNORD115 and the decrease in SNORD116 snoRNA levels were demonstrated [72]. It has been previously reported that the upregulation of SNORD115 due to the duplication of the region of mouse chromosome 7 that mirrors the human chromosome 15q11–13 duplication results in abnormal brain development. The observed change in SNORD115 level led to various psychic and behavioral aberrations typical of autism [73].

## 4. snoRNAs and Viral Diseases

Viral infection leads to the transcriptional activation of a vast group of genes that are related to the innate immune response genes [74, 75]. The activity of the products of such genes is aimed at suppressing the processes of viral development, particularly replication and transcription of viral genome, translation of viral mRNAs, and virus assembly [76]. Noncoding RNA genes have been found among the group of genes that are overexpressed during activation of antiviral response, but their role has not been clearly established yet [77, 78]. Recently, the first data on upregulation of the series of snoRNA genes in virus-infected human cells have been obtained [79]. On the one hand, snoRNA can act as mediators of host antiviral response; on the other hand, the activity of regulatory RNAs can be utilized by viruses to evade innate immunity and complete their life cycle [80].

The first evidence of the existence of snoRNA encoded in viral genomes was obtained from the studies on herpesviruses. A box C/D RNA termed as v-snoRNA1 (viral small nucleolar RNA1) has been found in B lymphocytes infected with Epstein-Barr virus (EBV) [81]. This RNA was 65 nt long and encoded within the viral genome. Despite the fact that the viral box C/D RNA has C/D and C′/D′ pairs of boxes and contains short regions (9–11 nt) complementary to human 18S and 28S rRNAs, the further analysis did not reveal 2′-O-methylation of potential rRNA target nucleotides in infected cells [81]. Apart from the full-length box C/D RNA, a 24-nt RNA that represents a 3′-terminal fragment (41st to 64th nt) of the viral box C/D RNA, v-snoRNA1^24pp^, was found in cells infected with EBV. In EBV genome v-snoRNA1 is encoded in the antisense direction to 3′-UTR of DNA polymerase gene (*BALF5*). It has been shown that v-snoRNA1^24pp^ is able to interact with* BALF5* mRNA and induce specific hydrolysis of target mRNA in the region of complementarity. The levels of the viral box C/D RNA and its shortened form increase 30-fold in cells during the lytic phase of infection. It has also been established that the level of* BALF5* mRNA in infected cells is controlled by the viral microRNA miR-BART2 during transition from latent to lytic phase [82]. Therefore, it is reasonable to assume that activation of viral box C/D RNA expression and its processing to short regulatory form enables a more rapid decrease in a target mRNA level. Bioinformatic analysis has demonstrated that a cognate box C/D RNA is encoded within the genome of another *γ*-herpesvirus, rhesus monkey* Lymphocryptovirus* [81]. The presence of box C/D RNA in viral genome indicates that viruses are able to utilize snoRNA-dependent mechanisms for the regulation of their life cycle.

Many RNA viruses, especially (+)ssRNA viruses, encode their own RNA processing machinery capable of RNA cleavage and modification. Among RNA viruses there are members of the order Nidovirales, comprising such well-known human pathogens as coronaviruses that uniquely encode within their genome an endonuclease designated as NendoU. Endonuclease NendoU bears striking homology to cellular XendoU, poly(U)-specific endoribonuclease A from* Xenopus laevis*, involved in snoRNA processing (e.g., U16 and U86 snoRNA maturation) [83]. XendoU is known to be broadly conserved among Metazoa, but its homologues in human and most laboratory animals remain hypothetical. Severe acute respiratory syndrome coronavirus (SARS-CoV) NendoU is a component of the replicase-transcriptase complex, but whether its role is critically important for the viral life cycle remains to be determined.

Vast majority of noncoding RNAs are subjected to differential regulation in response to virus infection, yet the examples of changes in expression of snoRNAs in infected cells are very limited. Recently, the analysis of lung transcriptome in mice infected with SARS-CoV or influenza A virus has revealed differential expression of 30 small RNAs overlapping with annotated snoRNAs. Two putative snoRNAs differentially regulated by respiratory virus infection were found in* GAS5* locus [84]. Intriguing,* GAS5* intronic snoRNAs U44, U76, and U78 were found to be upregulated in human cells infected with chikungunya virus [79]. It can be speculated that* GAS5* intronic snoRNAs may be involved in the regulation of T-cell growth [85].

The hypothesis that viruses are able to involve host snoRNAs in the regulation of stability and functionality of their RNAs is supported by the studies demonstrating that viral RNAs contain a variety of modified nucleotides [86, 87]. Moreover, it has been established that viruses are capable of utilizing other pathways in order to regulate the modification of their own mRNAs and evade host immune system [88–90]. In order to determine the role of snoRNAs in viral infections further research is needed.

## 5. Change in snoRNA Level in Mammalian Cells Subjected to Stress and Drugs

Changes in the level of snoRNAs circulating in body fluids were shown to be induced by different stress factors. At the scale of the whole organism any trauma is considered to be stress causing shift in homeostasis. Two box C/D snoRNAs U48 and U38 were found to be significantly overexpressed in serum of patients with anterior cruciate ligament (ACL) injury [91]. Moreover, U38 snoRNA expression level was reliably elevated in patients with cartilage damage associated with ACL injury. The snoRNA U38 may therefore serve as a biomarker for early diagnostics of osteoarthritis following joint injury.

Changes of snoRNA level can also occur in mammalian cells exposed to chemical stress factors. It has been demonstrated that Chinese hamster ovary (CHO) cells contain increased level of three box C/D snoRNAs—U32a, U33 and U35a, when exposed to fatty acids [92]. These snoRNAs are encoded within different introns of the ribosomal protein L13a (RPL13a). Under such conditions box C/D snoRNAs accumulate in cytoplasm, but not nuclei, thus suggesting a new cytoplasmic function of small nucleolar RNAs. Fatty acids are known to be toxic to mammalian cells. Lipotoxicity is characterized by initiation of oxidative stress, endoplasmic reticulum (ER) dysfunction, and can further lead to proinflammatory processes and apoptotic cell death [93, 94]. First it has been demonstrated that* RPL13a* is largely involved in activation of palmitate-induced cell death. Saturated fatty acids (myristate, palmitate, and stearate), unlike less toxic, unsaturated fatty acids (palmitoleate and oleate), have been reported to induce U32a, U33, and U35a snoRNA overexpression [92]. Despite the upregulation of snoRNA, there was no detectable change in 2′-O-methylation level of rRNAs. Specific downregulation of the reported box C/D RNAs protected cells from lipotoxicity. It is also notable that the suppression of U32a, U33, and U35a box C/D RNA genes in cells resulted in inhibited accumulation of H_2_O_2_-derived reactive oxygen species, thus implicating the participation of these snoRNAs in a common mechanism of cellular response to oxidative stress.* In vivo* experiments showed that administration of lipopolysaccharides induces U32a, U33, and U35a snoRNA expression in mouse liver [92]. Thus, small nucleolar RNAs are capable of participating in the regulation of cellular response to external stress and controlling activation of apoptosis.

Tamoxifen is one of the major drugs used for the hormonotherapy of breast cancer. It serves as an estrogen receptor antagonist and induces breast cancer cell death by apoptosis [95]. However, there are breast cancer types not susceptible to tamoxifen. Global analysis of the transcriptome in tamoxifen-sensitive and tamoxifen-resistant breast cancer cells revealed differences in snoRNA pattern between the two types of cancer cells [96]. Such data suggest that small nucleolar RNAs play a prominent role in sustaining drug-resistance in cancer cells, where targeted regulation of snoRNA expression may increase their sensitivity to the therapy.

Tenofovir disoproxil fumarate (TDF) is a nucleotide-derived inhibitor targeted to HIV reverse transcriptase. It is allowed for administration to HIV-infected patients as part of complex antiretroviral therapy. Patients under tenofovir treatment are known to experience bone tissue loss. In a recent study Grigsby et al. have profiled gene expression in primary murine osteoclasts [97]. The research demonstrated that TDF treatment causes downregulation of three genes—*GNAS*,* GOT2* and* SNORD32a*. It was proposed that, being combined with other cellular malfunctions, change in the snoRNA level induced by the drug administration can lead to aberrant rRNA biogenesis and induce cell death [97].

The data presented suggests that alterations in snoRNA expression profile can modulate the sensitivity of human cells to drugs.

## 6. Conclusion

RNA posttranscriptional modification and control of mRNA stability and translation are important parts of gene expression regulation in human cells. Small nucleolar RNAs and their functional fragments play a considerable role in these processes: they guide modification of rRNA and snRNA nucleotides, influence alternative splicing of complementary pre-mRNAs, and control translation and stability of mRNAs through RISC-dependent pathway. Disruption of snoRNA expression can be caused by both external factors and intracellular signal cascades and result in physiological changes on a cellular level, organ dysfunctions, and various diseases. The structure of snoRNAs, their expression pattern, and localization within cells have regulatory significance and are considered diagnostic markers of pathology. The insight into snoRNA expression and the mechanisms of their functioning will provide new possibilities for the development of diagnostic systems and novel therapeutic approaches for human diseases.

## Abbreviations

snoRNAs: Small nucleolar RNAs
rRNAs: Ribosomal RNAs
snoRNP: Small nucleolar ribonucleoprotein
SAM: S-Adenosylmethionine
SAH: S-Adenosylhomocysteine
scaRNAs: Small Cajal body-specific RNAs
snRNAs: Small nuclear RNAs
sdRNAs: SnoRNA-derived RNAs
NSCLC: Nonsmall cell lung carcinoma
SCC: Squamous cell carcinoma
AML: Acute myeloid leukemia
ALL: Acute lymphoblastic leukemia
AC: Adenocarcinoma
PWS: Prader-Willi syndrome
psnoRNAs: Processed snoRNAs
v-snoRNA1: Viral small nucleolar RNA1
EBV: Epstein-Barr virus
SARS-CoV: Severe acute respiratory syndrome coronavirus
ACL: Anterior cruciate ligament
CHO: Chinese hamster ovary
ER: Endoplasmic reticulum
TDF: Tenofovir disoproxil fumarate
HIV: human immunodeficiency virus.
